# Initial hospitalization with rigorous bed rest followed by bracing and rehabilitation as an option of conservative treatment for osteoporotic vertebral fractures in elderly patients: a pilot one arm safety and feasibility study

**DOI:** 10.1007/s11657-018-0547-0

**Published:** 2018-11-23

**Authors:** Tetsuya Abe, Yosuke Shibao, Yosuke Takeuchi, Yuki Mataki, Kuniaki Amano, Shigeru Hioki, Kousei Miura, Hiroshi Noguchi, Toru Funayama, Masao Koda, Masashi Yamazaki

**Affiliations:** 1 Department of Orthopaedic Surgery, Faculty of Medicine, University of Tsukuba, 1-1-1, Tennodai, Tsukuba-city, Ibaraki 305-8575 Japan; 2 Department of Orthopaedic Surgery, Tsukuba Central Hospital, 1589-3, Kashiwada-cho, Ushiku-city, Ibaraki 300-1211 Japan

**Keywords:** Osteoporotic vertebral fracture, Elderly, Bed rest, Conservative treatment

## Abstract

**Summary:**

We assessed the safety and feasibility of a unified conservative treatment protocol for osteoporotic vertebral fractures in the elderly patients with a 24-week follow-up. Our results showed that initial hospitalization with rigorous bed rest followed by a rehabilitation program using a Jewett brace was safe and feasible in managing patients.

**Purpose:**

The purpose of this study was to prove the safety and feasibility of a unified conservative treatment protocol, which included initial hospitalization with rigorous bed rest followed by a rehabilitation program with Jewett brace for osteoporotic vertebral fractures (OVFs) in the elderly patients with a 24-week follow-up.

**Methods:**

Between April 2012 and Mach 2015, one hundred fifty-four patients met the eligibility for this study. Radiological findings at the 3-week, 6~8-week, 24-week assessment were evaluated. Among these, 11 patients underwent early surgery within the first 2 weeks after admission and 19 patients lost follow-up. Therefore, 124 patients were assessed at the final follow-up visit.

**Results:**

The average vertebral instability in all the present series was 4.9 ± 4.8° at 3-week, 2.9 ± 3.5° at 6~8-week, and 1.8 ± 3.0° at 24-week follow-up visit. Delayed union was observed in 16 patients on the 24-week follow-up visit. Therefore, the present conservative treatment protocol resulted in bony union in 98 out of 124 patients (79.0%, per protocol set analysis) and 98 out of 154 patients including drop-out (63.6%, intention-to-treat analysis). There was no severe adverse event related to initial bed rest. The vertebral instability at 3-week assessment was significantly higher in the delayed union group when compared with that in the union group. Univariate analyses followed by multivariate logistic regression analysis revealed that T2-weighted image of confined high intensity on MRI and having more than 5° of vertebral instability on dynamic X-ray at 3-week assessment are the independent risk factors for delayed union of conservative treatment in the present series.

**Conclusions:**

Our results showed that initial hospitalization with rigorous bed rest followed by a rehabilitation program using a Jewett brace was safe and feasible. Therefore, the present conservative treatment protocol can be one of the acceptable treatment options in managing OVF patients.

## Introduction

Osteoporotic vertebral fractures (OVFs) are a well-known disease with common occurrence in elderly patients. OVF is conventionally treated with conservative management, comprising bed rest, analgesic therapy, and wearing orthosis [6, 9, 10, 12–14, 16, 18]. Current treatment for the majority of elderly patients with an OVF is an out-patient in the world. The patients in the majority seem to lead to a successful resolution of symptoms. When bone union is not achieved within 6 months, patients continue to have intractable pain and a risk of developing neurological deficits due to spinal instability related to the delayed-/non-union [7, 11, 23, 24]. A recent prospective multicenter study showed that conservative treatment for elderly patients with OVFs carries a risk of delayed union and non-union of 13.5% [23]. They revealed that presence of the middle column injury and some of intensity changes on T2-weighted magnetic resonance images (MRI) were significant risk factors of delayed-/non-union. However, a standardized conservative management for OVFs has not been established. Kishikawa [13] reported that initial bed rest for 2 weeks plays an important role in preventing vertebral collapse and reducing pain in elderly patients with OVFs during short-time follow-up; however, their report lacks longer follow-up results. In addition, there is no report on conservative treatment for OVFs to prevent delayed paralysis due to non-union (pseudarthrosis) of the fractured vertebra.

For the very first step to prove the efficacy of the present conservative treatment protocol, we perform one arm feasibility study. The purpose of this study was to prove the safety and feasibility of a unified conservative treatment protocol, which included initial hospitalization with rigorous bed rest in 2 weeks followed by a rehabilitation program with a Jewett brace for OVFs in the elderly patients with a 24-week follow-up.

## Methods

This is a prospective single-centered, cohort study to assess the safety and feasibility of the present conservative treatment protocol for OVFs in elderly patients in Japan.

The inclusion criteria were (1) age of 60 years or older, (2) the presence of acute onset back and/or low back pain due to single-level vertebral fracture caused by minor or non-traumatic events, and (3) agreement for admission to treat back pain. Inclusion criterion (2) was set because Japanese national survey revealed that OVFs were increased in population over 60 years old [8]. The exclusion criteria were pathological fracture due to malignancy, being non-ambulatory before the onset of the fracture, severe dementing disorder to impede bed rest, and refusal of admission to treat back pain.

The present conservative treatment protocol was as follows: all patients with the suspicion or the diagnosis of OVF were admitted. The fractures were confirmed by MRI, computed tomography (CT) scan, and dynamic X-ray in standing and supine position [19]. From these imaging examinations, patients with definitive diagnosis of OVF were forbidden to sit up even for dietary intake and evacuation during the initial 2 weeks [13]. All patients were instructed to lie down with the lateral position on bed. Patients were allowed about rolling over to semi-Fowler position on 20~30° of head elevation to keep the patient’s kyphosis, so that the patients’ back fit well alongside to the surface of the bed. Following the confirmation of decrement of their back and/or low back pain, which was assessed with at least 50% decrement of back pain evaluated with visual analogue scale (VAS), 2 weeks after hospitalization, they were allowed to get out of bed for rehabilitation program, which mainly includes standing up, walking, muscle training of lower extremities, wearing a ready-to-use Jewett brace (Kobayashi medical, Shimane, Japan), which restricts lumbar flexion and could be worn even if the patients had severe thoracolumbar kyphosis. All the patients were instructed to wear the orthosis for 12 to 24 weeks until the pain and the vertebral instability disappeared. The patients were allowed to take non-steroidal anti-inflammatory drugs according to the severity of pain. The present study was performed to prove the feasibility of the present conservative therapy protocol, because there was no consensus on the duration of bed rest, optimal rehabilitation program, and timing of initiation of rehabilitation for OVFs [15, 20].

**Fig. 1:**
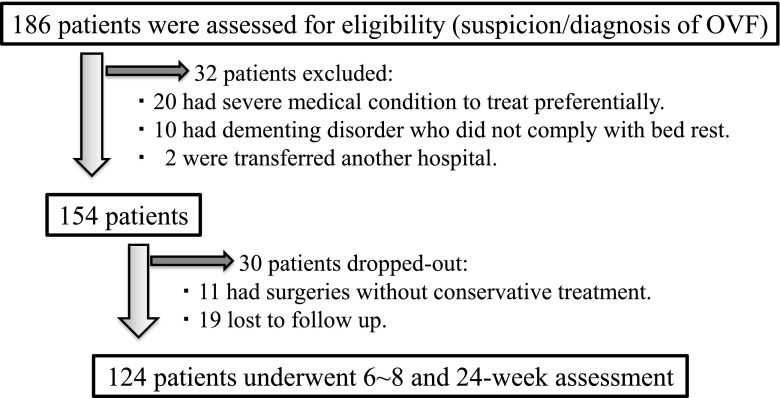
Flow diagram for patient enrollment and follow-up of the 186 OVF patients admitted, 32 patients were excluded. Therefore 154 patients were included to this study. Among them, 11 patients underwent early surgery within first 2 weeks after admission and 19 patients lost follow-up for 6~8-week and/or 24-week assessment. Finally, 124 patients were analyzed at 24-week follow-up visit

Between April 2012 and Mach 2015, 186 consecutive patients of OVFs were considered as candidates for the present protocol. Thirty-two patients were excluded from the present protocol. Of these, 20 patients had severe medical condition to treat preferentially. Ten patients were impossible to apply bed rest because of their dementing disorder. Two patients transferred to another hospital. As a result, 154 patients met the eligibility of the present protocol. Among these, 11 patients underwent early surgery within the first 2 weeks after admission and 19 patients lost follow-up for 6~8-week and/or 24-week assessment. Therefore 124 patients including 18 males and 106 females were assessed for final follow-up results (Fig. 1). The improvement of back/low back pain and dynamic X-ray was assessed 3 weeks, 6~8 weeks, and 24 weeks after enrollment.

### Radiological evaluations

**Fig. 2:**
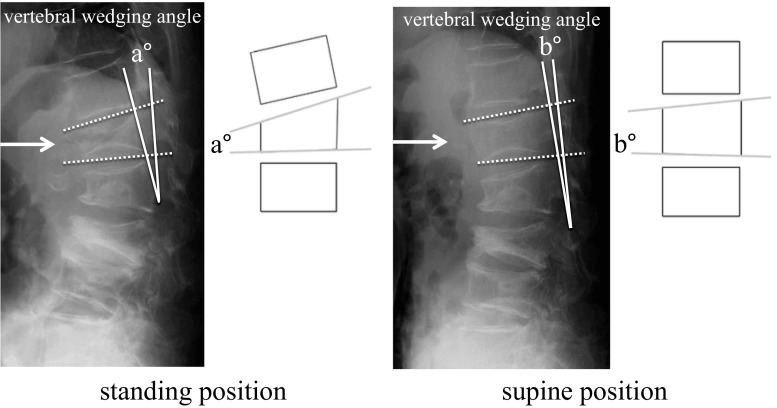
Defining vertebral instability. It was calculated as the difference (absolute value) of the vertebral wedging angle between standing (**a**) and supine (**b**) position. The difference in the angle formed by drawing a perpendicular line along the superior and inferior endplates of the fractured vertebra and measuring the intersection looking at the difference between this measurement in a supine position and a standing position

The vertebral instability was calculated as the subtraction of the vertebral wedging angle, which was the angle between the superior and the inferior endplates, of standing from supine position (Fig. 2). The dynamic X-ray in this study had consistency between measures at baseline and 24-week assessment; CT scan was used for precise evaluation of vertebral fractures including middle and posterior column of the vertebrae. T2-weighted intensity changes in the MRI of the fractured vertebra were divided into five types including diffuse low, diffuse high, confined low, confined high, and no intensity change according to Tsujio’s report [23].

Twenty-four weeks after enrollment, vertebral instability within 5° was defined as union, because 5° was roughly detectable vertebral instability using dynamic X-ray by two observers in our preliminary study (*n* = 50). A cutoff value of 5° was 98% of sensitivity and 98% of specificity. Delayed union was defined as vertebral instability of more than 5° and/or the detection of intra-vertebral cleft on the lateral X-ray in supine position [11].

### Outcome measures

In the present study, patients with OVFs defined as delayed union at 24-week assessment were followed over 6 months whether delayed paralysis occurs or not. Complications and length of hospital stay were also assessed.

Ambulatory status before the onset of back pain, which was self-reported by patients with the ambulatory status 1 month before admission, at the discharge from our institution and at 24-week follow-up visit was assessed according to the functional mobility scale with walking distance of 50 m, which was classified into six levels as follows: level 1, wheelchair; level 2, use of regular front or reverse walker; level 3, walk with crutches without help; level 4, one crutch or 1–2 canes/poles/walking sticks; level 5, independent walking on level surface; level 6, independent walking and running on all surfaces [4]. We defined patients with ambulatory status from level 3 to 6 as “independent” and patients with ambulatory status levels 1 and 2 as “dependent.”

Complications during the hospitalization were collected from clinical records.

### Statistical analyses

Overall ratio of bony union was analyzed by two different strategies of analyses, in both 154 patients who met eligibility criteria (intention to treat; ITT) and 124 patients excluded early surgery cases and drop-out (per-protocol-set; PPS).

The patients with final 24-week follow-up data (PPS) were divided into two groups that comprised the union group and the delayed union group. Clinical outcomes and radiological findings were compared between both groups. The differences between the two groups of 3-week, 6~8-week, and 24-week results were assessed using the Student’s *t* test, Wilcoxon rank sum test for continuous variables, or the *χ*^2^ test for categorical variables. A *p* value of less than 0.05 was considered statistically significant.

To clarify the risk factors for delayed union of the present conservative treatment protocol, statistical analyses were performed. Correlation between delayed union and all the factors possibly related to the delayed-/non-union including age, sex, body mass index, history of trauma, initial leg pain, visit by ambulance, previous osteoporosis medication, past OVF history, and use of steroids was assessed with univariate analyses, and factors that showed a *p* value of less than 0.1 were selected for further analyses. Next, the selected factors were served for logistic regression analyses using a stepwise method to determine which independent factors were significantly associated with delayed union of the present conservative treatment protocol. Values are expressed as mean ± standard deviation (SD). Odd ratio (OR) for the incidence of failure and the 95% confidence intervals (CI) were calculated as an approximation of the relative risk estimates. All statistical analyses were conducted using SPSS software for Windows (version 10.0; SPSS, Inc., Chicago, IL).

## Results

In the present series, the mean age was 81.2 years (range, 60–98 years). The follow-up rate was 80.5%. One hundred ten patients (71.4%) were diagnosed within 2 weeks, 13 patients (8.4%) were within 2 months, and 1 patient (0.6%) was diagnosed over 2 months after the onset of their back and/or low back pain. Sixty-eight patients (44.2%) had history of trauma including falling as the onset of back pain. On admission, 46 patients (30.0%) were ambulatory and 78 patients (50.6%) were visited with emergency ambulance. Thirty-four patients (22.1%) had been treated with bisphosphonates and 2 patients (1.2%) with selective estrogen receptor modulator as a medication for osteoporosis. None had been treated with both parathyroid hormone and anti-RANKL antibody preparation on admission. In the present study, the medication prescribed kept through the 24-week assessment. Twenty-eight patients (18.2%) had past OVF history. The mean number of previous fractures was 2.2. Seven patients (4.5%) made use steroids because of their medical condition. The *t* score of bone mineral density (g/cm^2^) was 0.829 ± 0.202 in the spine, 0.625 ± 0.129 in the right femoral neck, and 0.639 ± 0.155 in the left femoral neck, respectively. Twenty patients (13.0%) had dementia, which did not prevent their inclusion into the present series because they tolerated well the rigorous bed rest for 2 weeks. The patients’ demographic data was shown in Table 1.

**Table 1:** Demographic data of patients with osteoporotic vertebral fractures (*n* = 154)

Sex (male: female)	22: 132
History of trauma	68 (44.2%)
Leg pain	7 (4.5%)
Visit by ambulance	46 (30.0%)
Previous osteoporosis medication	34 (22.1%)
Past history of OVF	28 (18.2%)
Use of steroids	7 (4.5%)
Dementing disorder	13 (13.0%)
Lost to follow-up (drop-out)	19 (12.3%)

*OVF*, osteoporotic vertebral fracture

**Fig. 3:**
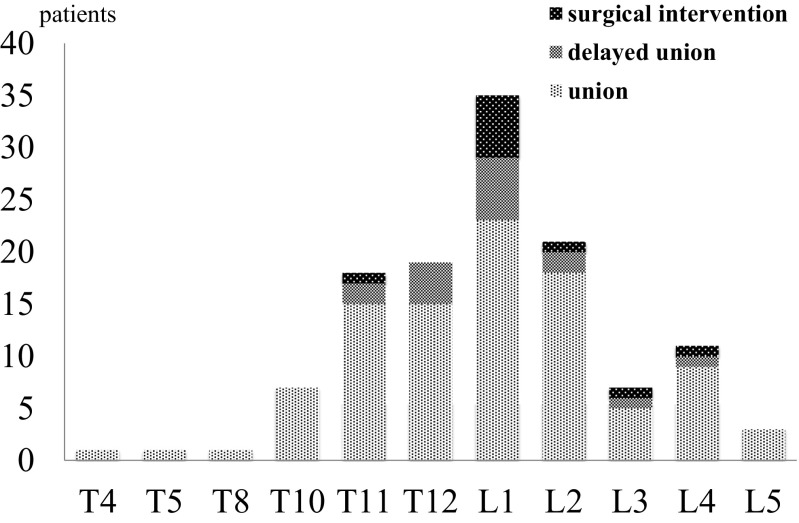
Clinical outcomes at 24-week assessment according to the affected levels (*n* = 124). All patients had vertebral fracture at a single level. Six patients had leg pain due to vertebral fractures and its instability at L2 in 1 patient, L4 in 4 patients, and L5 in 1 patient. Osteoporotic vertebral fracture and delayed union mainly occurred at thoracolumbar levels (T11-L1) in 73 patients (58.9%) and 12 patients (75%), respectively

The affected vertebrae were T4, T5, and T8 in 1 patient; T10 in 7 patients; T11 in 18 patients; T12 in 19 patients; L1 in 35 patients; L2 in 21 patients; L3 in 7 patients; L4 in 11 patients; and L5 in 3 patients (Fig. 3). Delayed union was observed in 16 patients on the 24-week follow-up visit. Therefore, the present conservative treatment protocol resulted in bony union in 98 out of 124 patients (79.0%, PPS analysis) and 98 out of 154 patients including surgical cases and drop-out (63.6%, ITT analysis). However, the 16 patients with delayed union were treated conservatively with prolonged bracing for 6 to 12 months after the present study protocol. Finally, 14 out of these patients who did not exhibit bone union at 24-week assessment had progressed to exhibit bone union 1-year follow-up. In the surgical intervention group, 7 patients were treated with Balloon Kyphoplasty (BKP) and 3 patients were treated with posterior pedicle screw fixation combined with vertebroplasty using auto bone grafting to reduce not only the intra- but also inter-vertebral instability. These results were shown in Table 2. Only one patient who was treated with BKP had repeated surgery because of cement instability [17]. They had no subsequent vertebral fracture after surgery [1].

**Table 2:** Surgical intervention group (*n* = 10)

Days after admission	Mean 39.6 days (28~80 days)
Vertebral instability	Mean 10.8°(5.7~17.7°)
MRI (T2-weighted intensity)	
Confined high	6 cases
Confined low	2 cases
Diffuse low	2 cases
Middle column injury (CT)	
+	8 cases
−	2 cases
Surgeries	
BKP	7 cases
VP + PLF	3 cases
Ambulatory status	
Improve	1 case
Worsen	3 cases
No change	6 cases

*BKP*, Balloon Kyphoplasty; *VP*, vertebroplasty; *PLF*, posterolateral fusion

In all the patients (PPS analysis), the vertebral instability was measured in standing vs supine position from 3-week to final follow-up assessment. Initial dynamic X-ray evaluation could not be performed in a half of these, because of their intractable pain. Approximately a half of initial dynamic X-ray evaluation was performed in sitting and supine position because patients could not stand due to pain. The average vertebral instability in the present series was 4.9 ± 4.8° at 3-week, 2.9 ± 3.5° at 6–8-week, and 1.8 ± 3.0° at 24-week follow-up visit. Therefore, the evaluation of sagittal alignment of the lumbar spine was not included because initial whole X-rays were not taken in the present study [5]. Six patients who had vertebral fracture (at L2, L4, and L5) and complained leg pain improved their symptoms during initial 2-week bed rest. No patients exhibited neurological deficits. In the union group, the vertebral instability was 4.1 ± 4.3° at 3-week, 2.3 ± 2.8° at 6–8-week, and 1.2 ± 2.5° at 24-week assessment. In the delayed union group, the vertebral instability was 9.6 ± 4.9° at 3-week, 6.8 ± 4.6° at 6–8-week, and 4.9 ± 3.5° at 24-week assessment. The vertebral instability at 3-week, 6~8-week, and 24-week assessment was significantly higher in the delayed union group when compared with that in the union group (*p* < 0.05). According to CT scans, 49 of 124 patients (39.5%) exhibited middle column injury of the fractured vertebra. In this study, the middle column injury was not correlated with bone union at 24-week assessment (Table 3). T2-weighted MRI image showed confined low-intensity lesions in 56 patients (45.2%), diffuse low-intensity lesions in 23 patients (18.5%), confined high-intensity lesions in 25 patients (20.2%), diffuse high-intensity lesions in 3 patients (2.4%), and no intensity change in 5 patients (4%).

**Table 3:** Risk factors for failure of conservative treatment of osteoporotic vertebral fractures

Univariate analysis	*p* value (^#^: *p* < 0.1)
Age	0.15
Sex	0.7
BMI	0.097
History of trauma	0.026^#^
Leg pain	0.85
Visit by ambulance	0.89
Previous osteoporosis medication	0.45
Past OVF history	0.56
Use of steroids	0.55
Vertebral instability on X-ray	0.0002^#^
T2WI confined high on MRI	0.0001^#^
Middle column injury on CT	0.25
Stepwise logistic regression	*p* value (*: *p* < 0.05)
History of trauma	0.096
Vertebral instability (X-ray)	0.0024*
T2WI confined high (MRI)	0.0016*

*BMI*, body mass index; *CT*, computed tomography; *MRI*, magnetic resonance imaging; *T2WI*, T2-weighted images

Body mass index, history of trauma, vertebral instability, and T2-weighted image of confined high intensity on MRI showed *p* value of less than 0.1 in the univariate analyses, then those factors were served for logistic regression analyses using a stepwise method to determine which independent factors were significantly associated with delayed union. Multivariate logistic regression analyses revealed that T2-weighted image of confined high intensity on MRI (OR, 4.2; 95% CI, 2.0–8.8; *p* = 0.0001) and having more than 5° of vertebral instability on dynamic X-ray at 3-week assessment (OR = 1.3, 95% CI = 1.0–1.4, *p* = 0.0002) are the independent risk factors for delayed union after conservative treatment in the present series.

The ambulatory status before injury was classified as level 1 in 6 patients, level 2 in 24 patients, level 3 in 4 patients, level 4 in 21 patients, and level 5 in 59 patients according to the functional mobility scale. Only 2 patients lost the ambulance because of their dementing disorder. The independent and dependent ambulatory status on admission was seen in 84 and 30 patients, respectively. The independent and dependent ambulatory status on both at discharge from our institution and 24-week follow-up visit was seen in 66 and 48 patients, respectively. The number of patients with dependent ambulatory status was significantly increased after OVFs (*p* = 0.012). However, there was no difference between the union group and the delayed union group.

The length of stay in hospital was a mean of 54 ± 29 days. A 2-week plus hospital stay was within 2 weeks in 25 patients (21.9%), 2~4 weeks in 41 patients (40%), and over 4 weeks in 48 patients (42.1%), respectively. The mean stay in hospital of the union group and the delayed union group was 52.2 ± 28.1 days and 64.4 ± 32.9 days, showing no statistical difference between both groups. Further investigation would be warranted to estimate the cost of a 2 week plus stay in hospital for bed rest versus out-patient management [1, 17].

As for complications, urinary tract infection was recorded in 10 patients, pneumonia was recorded in 5 patients, and ileus was recorded in 1 patient during initial hospitalization. There was no difference in the occurrence of complications between the union group and the delayed union group. Severe life-threatening complications possibly related to bed rest including deep vein thrombosis and pulmonary embolism did not occur in the present series. There was no patient showing delayed paralysis in this study.

## Discussion

The present results showed that initial hospitalization with rigorous bed rest followed by rehabilitation program with a Jewett orthosis was accomplished in 86.7% of patients (124 out of 143 patients, ITT analysis) with OVFs without severe adverse events related to bed rest. Bone union was achieved in 80% of patients (98 out of 124 patients, PPS analysis) at 24-week assessment with the present conservative treatment protocol, whereas 16 out of 124 cases (14%) resulted in delayed union.

Tsujio reported that the confined high-intensity lesions on MRI T2-weighted images of fractured vertebra were a risk factor of the non-union [23], which agrees with our findings. The X-ray finding of vertebral instability and confined high-intensity lesions on MRI T2-weighted images indicate the loss of continuity of trabecular structure of the part of the fractured vertebra, possibly affecting bony union.

Delayed union should be avoided because it might cause intractable low back/lower extremity pain and delayed paresis, possibly requiring reconstructive spine surgery [21, 22], which could be too invasive for elderly patients with various comorbidities. In the present study, 24 patients (24.5%) in the union group were discharged within 30 days on admission, whereas only 1 patient (6.3%) was discharged within same periods. Further investigation would be warranted to estimate the cost of a 2-week plus stay in the hospital for bed rest versus out-patient management. Delayed union patients required prolonged conservative treatment with Jewett orthosis for additional 6 months. At present, there is no definitive conclusion about the optimal treatment for delayed union, even though most of the delayed union patients showed bone union 1 year after enrollment to the present study. Further exploration is needed to elucidate this issue.

The incidence of pseudarthrosis had been reported as 14 to 34.8% in the previous reports [9, 24]. Hoshino reported that the difference of initial treatment including brace type, hospitalization, and administration of bisphosphonates, and painkillers did not affect activities of daily living, pain, cognitive ability, or vertebral collapse at 6 months after the onset of OVFs in a multicenter study including 363 patients [9] and they also reported that the incidence of pseudarthrosis 6 months after onset of OVFs was 14%. Wu reported that OVFs with middle column injury exhibited significantly higher incidence of pseudarthrosis when compared with that of patients with anterior column injury alone [24]. It is impossible to directly compare the incidence of pseudarthrosis in those previous studies and in the present study because there is significant variation in the conservative treatment protocol and in the definitions of pseudarthrosis, delayed union, and failure of conservative treatment. The efficacy of brace application for the treatment of OVFs also remains unclear. The quality of studies examining the effectiveness of orthosis for the management of OVFs is generally limited [6, 12, 18]. Kim reported that the Oswestry Disability Index scores for the treatment of OVFs without a brace were not inferior to those with soft or rigid braces. Moreover, the improvement in back pain and progression of anterior body compression were similar among them in their randomized controlled study [12]. In addition, clinical outcomes seemed to be good in terms of preventing delayed paralysis due to non-union (pseudoarthrosis) of the fractured vertebra. The present conservative treatment protocol has several possible benefits for OVFs in elderly patients. Rigorous bed rest at an early phase of OVFs might reduce not only the progression of vertebral collapse [12] but also the onset of delayed paralysis [2, 21, 22]. However, the design of the present study was not a comparative study; we could not conclude the effect of rigorous bed rest on OVF. Next, optimal timing of surgical intervention could be determined by the patients’ response to the initial rigorous bed rest. In the present series, all the patients who required surgery due to their intractable back/lower limb pain underwent surgery just after the initial bed rest for 2 weeks. The surgical indication for patients who underwent early surgery was mainly based on the patients’ complaint not by true medical indication (e.g., progression of paralysis). We consider the present conservative treatment protocol as one of the screening tools to determine indication of VP/BKP [3], which is highly effective to reduce intractable pain in most OVF patients. However, we had no data about the cost-effectiveness in both outpatients and inpatients with rigorous bed rest. Further exploration is needed to elucidate the cost-effectiveness of the present protocol.

As for adverse events, it is an important concern that complications related to bed rest might increase if a rigorous bed rest is applied to the elderly population. That concern is one of the causes for the recommendation of early surgical intervention for OVFs in elderly. Contrary to our expectation, there was no apparent increase of severe adverse events related to bed rest in the present series. Sixteen patients (12.9%) had complications including urinary tract infection in 10 patients, pneumonia in 5 patients, and ileus in 1 patient. Whether those complications were caused by loss of their activity before admission or by bed rest is unclear. However, these complications were treated with antibiotics conservatively during initial hospitalization. The other possible adverse effects caused by bed rest are sarcopenia and functional decline. There are few reports on the factors associating with impairment of ambulatory status of patients with OVFs. Yagi [25] reported that the frequency of ambulatory status decline related to conservative therapy without initial rigorous bed rest was approximately 20 out of 60 patients (33%). Therefore our present data about the functional decline is not inferior to the previous ones. As for sarcopenia, unfortunately, we did not assess the muscle volume in the present study. Future investigation is needed to prove the influence of bed rest on muscle volume. Together with the fact that there was no patient showing delayed palsy in the present series, we thought that bed rest in the elderly patients have a potential benefit to avoid delayed-/non-union requiring invasive reconstructive spine surgery. The optimal duration of bed rest, optimal rehabilitation methods, and optimal initiation of rehabilitation should be determined by further investigation with control arms.

The present study was conducted in Japan, of which country that the cost for hospitalization is cheaper than that in the other country such as the USA because of the nationwide coverage by the national health insurance system in Japan. Therefore, the hurdle for hospitalization is much lower and the length of hospital stay can be longer in Japan compared with the other countries. The possibility that the specific insurance system in Japan might have influence to the present result should be kept in mind to interpret the present result.

## Conclusions

Our results showed that initial hospitalization with rigorous bed rest followed by a rehabilitation program using a Jewett brace was safe and feasible in managing patients with OVFs without severe adverse events related to bed rest.
